# Effectiveness of virtual team learning in entrepreneurship education: a survey study

**DOI:** 10.1007/s41959-022-00064-0

**Published:** 2022-03-31

**Authors:** Li Chen, Dirk Ifenthaler, Wenting Sun, Tao Xu, Guanghao Yan

**Affiliations:** 1 Business School, University of Mannheim, Mannheim, Germany; 2 Computer Science School, Humboldt Universität Zu Berlin, Berlin, Germany; 3 School of Innovation and Entrepreneurship, Tongji University, Shanghai, China; 4 College of International Business, Zhejiang Yuexiu University of Foreign Language, Shaoxing, China

**Keywords:** Virtual team learning, Teamwork, Taskwork, Information and communication technology, Entrepreneurship competence

## Abstract

This study examines the effectiveness of virtual team learning for entrepreneurship competence in the Chinese higher education sector. Related research on the effectiveness of virtual team learning is sparse, especially in the area of entrepreneurship education. We assumed four hypotheses to analyze two sorts of relationships: one between input, respondents’ demographics or characteristics, and mediators, namely virtual teamwork, virtual taskwork, information and communication technology; the other between mediators and output, thus the effectiveness of entrepreneurship education. An online survey was carried out to collect respondents’ perceptions of virtual team learning in entrepreneurship education from teamwork, taskwork, and information and communication technology aspects, considering respondents’ demographics or characteristics. By explaining factors of the team process, the findings show that virtual teamwork, taskwork, and information and communication technology positively affect the entrepreneurial outcome of virtual team learning. Additionally, individual characteristics, including gender, education degree, education field, entrepreneurial family history, and prior entrepreneurial experience have different effects on three elements of virtual teams. The applied model provides a holistic perspective on virtual team learning and explains the association between three sectors. These findings may provide an empirical basis for making decisions in the design and development of entrepreneurship learning and teaching offerings.

## Introduction

Entrepreneurship education (EE), in which educators and learners learn and teach entrepreneurial knowledge, skills, and mindset (Man et al., 2002; Morris et al., 2013), originated in the USA and become mainstream in and outside business schools throughout the world. Entrepreneurship competence (EC) includes sub-competencies from marketing, business, management, economics, and law, as well as other related fields. In comparison with the competence classification by its level, where the focus is on the behavior and process of starting a business, educators and learners can efficiently teach and acquire EC related to varied fields. Many studies have shown that learners can acquire entrepreneurial competence (Blenker et al., 2014; Marques & Albuquerque, 2020; Nabi et al., 2018). Findings focusing on outcome or performance of online learning in the context of EE, however, is sparse (Liguori & Winkler, 2020), especially virtual team learning applied in EE in Higher Education Institutes (HEIs) as well as online learning combined with virtual team learning for supporting EE peer’s connectedness (Parrish et al., 2021).

Team learning methods foster the competence of problem-solving and identification of opportunities, exceeding entrepreneurial knowledge and skill acquired in HEI EE courses. Team learning is the favored EE learning and teaching method of late, followed by poster reports and engaging students in activities (Balan & Metcalfe, 2012; Pittaway & Cope, 2007). Team behavior affects entrepreneurship learning outcomes and moderates the correlation between entrepreneurship learning motivation and performance (Hytti et al., 2010). Furthermore, team activities provide entrepreneurship cognition, social networks, and practical experience, all of which are essential for (would-be) entrepreneurs (Man, 2007). Besides, team learning, except for face-to-face EE, is a critical method in online and blended EE. In line with the launch of the *action plan of education informatization 2.0* (MEPRC, 2018), Chinese educators and policymakers have started integrating information technology and team learning methods—virtual team learning in an online and blended learning environment. In the previous research, the concept of a virtual team has mainly been discussed in the context of an educational organization or workplace (Chumg & Huang, 2021; Elyousfi et al., 2021) and seldom in the field of formal education. Besides, the virtual team learning method is mainly adopted in the online learning environment (Ismailov & Laurier, 2021; Wen et al., 2015). With the application of the virtual team learning method in a complex and ambiguous blended or face-to-face EE course, it is necessary to investigate current developments. Further, we applied the input-mediator-output model to this survey and considered respondents’ demography or characteristics (input), teamwork, taskwork, and information and communication technology (mediators), leading to the EC of virtual team learning (output).

## Background

### Entrepreneurship competence

EE provides courses and activities to develop learners’ entrepreneurial knowledge, skills, and attitudes or views toward creating and operating a venture successfully, developing a career, or having a valuable life (Fejes et al., 2019). The competence of enterprises differs slightly from the terms of the management competence, with the former emphasizing identification of venture creation opportunity and the latter focusing on running a venture. In other words, EC focuses on identifying an opportunity for venture creation. The components of competencies of entrepreneurship are discussed from various aspects. With the European Commission’s definition of entrepreneurship, the framework of EC (EntreComp) was published in the year of 2016 and slightly modified in 2018. The framework is a broad interpretation that contains idea and opportunity competence (e.g., opportunity recognition and assessment), mobilizing resource competence, and competence in taking action to acquire financial, social, and cultural value. Educators, entrepreneurs, and stakeholders need to modify the theoretical framework based on specific situations and educational activities. This research reviewed an additional 11 papers published between 2001 and 2019 and compared them to EntreComp to understand the specific competencies of entrepreneurship.

Morris et al. (2013) argued that entrepreneurial competencies involve entrepreneurship and a series of basic business competencies. Mitchelmore and Rowley (2013) explored EC into entrepreneurial and management competence. Man et al. (2002) reviewed 12 pieces of literature related to the competencies, considering six areas, opportunity, relationship, conception, organization, strategy, and commitment in particular. In detail, conceptual competencies are reflected in entrepreneurs’ behavior, decision-making, risk-taking, and innovation. Commitment competencies refer to driving entrepreneurs to the firm’s moving forward. Halberstadt et al. (2019) identified five key competencies. Three of them, social competence, namely networks with various stakeholders, are similar to EntreComp. Business competence involves mobilizing resources and adopting firm strategies. Industry-specific competence is closely related to the exploitation of opportunities. Santos et al. (2019) focused on team entrepreneurial competence aspects, encompassing both team and individual levels. They separated innovation and creativity into two individual competencies. Lilleväli and Täks (2017) differentiate in entrepreneurs’ competencies between occupation (entrepreneurship) and the individual (entrepreneur). The former consists of both conceptual and operational elements, while the latter is composed of meta-competence, effectiveness, and social competence. Oosterbeek et al. (2008) separated EC into six traits and skills (market awareness, creativity, and flexibility), while the competencies: *the need for autonomy, the need for power, and flexibility* were not shown in the EntreComp. Akhmetshin et al. (2019) emphasized the division of EC into traits and skills/ competence. In addition, they focused on entrepreneurial knowledge and experience. Sánchez (2013) focused on personality traits, namely self-efficacy, proactiveness, and an inclination toward risk-taking. Mitchelmore and Rowley (2010) built a framework of entrepreneurial competence which narrows down EC as the identification of entrepreneurial opportunities, including management competence, human relations competencies, and conceptual/relationship competencies. Morris et al. (2013) outlined 13 specific competencies. In comparison with Man et al. (2002), Lans et al. (2014) adopt a broader interpretation of entrepreneurial competence, including financial and economic literacy, for example, and this classification has been structuralized.

The eleven literature sources were compared to the EntreComp framework proposed by the European Commission. Table 1 shows the calculated ratio of 15 sub-competencies.

**Table 1:** Entrepreneurship competence of 12 pieces of literature

Competence area	Sub-competence	Literature source												Ratio (%)
		1	2	3	4	5	6	7	8	9	10	11	12	
Idea/opportunities	Opportunity recognition	+	+	+	+	+	+	+	+	+		+	+	92
	Creativity	+	+			+	+	+		+	+	+	+	75
	Vision*	+		+	+	+					+		+	50
	Valuing ideas*	+		+								+	+	33
	Sustainable*					+	+						+	25
Resources	Perseverance	+			+	+	+	+			+		+	58
	Mobilizing resource/organization	+		+	+		+				+		+	50
	Finance			+		+	+						+	25
	Mobilizing human* resource		+	+	+	+	+	+					+	58
	Self-efficacy				+	+		+	+		+	+	+	58
Into action	Learning through experience			+			+						+	25
	Social network	+	+	+		+		+		+	+	+	+	75
	Common with ambiguity and risk	+	+			+	+	+	+				+	58
	Management		+	+	+	+	+			+		+	+	67
	Taking the initiative	+	+			+			+	+		+	+	58

Due to the lack of practical experience when applying EntreComp (Czyzewska & Mroczek, 2020), this study developed the framework further. We did not consider the low ratios of *ethical & sustainable thinking* in this paper. *Valuing ideas* was combined with *opportunity recognition. Mobilizing others* and *mobilizing resources* were combined as *mobilizing resources*. *Vision* is not considered in this study. Based on the classification of competence introduced by Lilleväli and Täks (2017) and Akhmetshin et al. (2019), this study incorporated 11 sub-competencies in two sections: *position* and *personality traits*. *Position* includes *opportunity recognition, mobilization of resources, taking the initiative*, *finance, learning through experience, social network,* and *management.* The *personality traits* section looks at *perseverance, self-efficacy, coping with ambiguity& risk actively,* and *creativity*.

### Virtual team learning

Virtual teams are organizations that use mainly information and communication technology (ICT) to facilitate the completion of tasks (Maznevski & Chudoba, 2000) in educational environments and workplaces (Ismailov & Laurier, 2021; Laitinen & Valo, 2018). Here, the definition of a virtual team is synonymous with an online, remote, or distance team/group. Virtual team learning is introduced into a virtual setting to promote learners’ socialization through asynchronous/synchronous and verbal/nonverbal methods that use email, video, audio, and multimedia social networking software. Trainee or potential entrepreneurs learn skills from experienced entrepreneurs, not only through listening, but also by applying and acting on their advice (Ratten, 2020). The virtual team enables learners and employees who are not in close geographical proximity to each other, to be connected (Bell & Kozlowski, 2002). Although students have returned to school since the Corona virus lockdown, educators, policy-makers, and stakeholders still need to improve virtual learning, for any future crisis (Ratten, 2020).

Teams or virtual teams reflect a complex system (Ilgen et al., 2005), featuring three central elements: *teamwork*, *taskwork*, and *ICT* (Holtkamp et al., 2015; Müller & Antoni, 2020; Warkentin & Beranek, 1999). In the current study, the application of ICT for communication and idea exchange is a basic requirement for a teammate (Holtkamp et al., 2015). Because online and blended learning environments lack opportunities for connection and communication, *ICT* is applied in a virtual team for communication and task completion in the process of learning and teaching. Facebook (Pittaway & Edwards, 2012), Twitter (Price et al., 2018), and podcast (Marques & Albuquerque, 2020), for example, were all utilized in EE courses. In research, taskwork and teamwork are slightly different concepts, although scholars usually take them as two similar facets of the team and analyze them together. In interaction, however, there is a difference between taskwork and teamwork (Nissen et al., 2014). In simple terms, teamwork is “collaboration” and taskwork refers to “cooperation” (Crawford & LePine, 2013). Specifically, taskwork focuses on task activities and devices used to complete a specific task, while teamwork emphasizes collaboration, interaction, or relationship strengths and weaknesses (Fisher, 2014; Müller & Antoni, 2020).

**Fig. 1:**
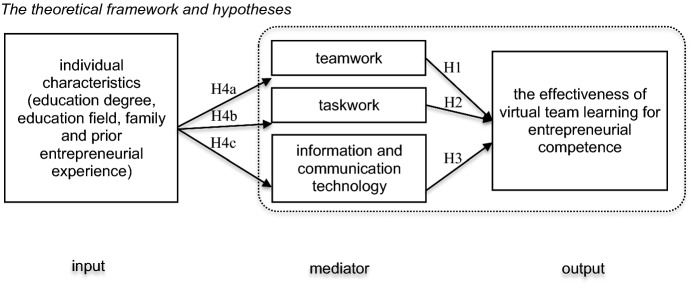
The theoretical framework and hypotheses

Based on the theory of team compilation and performance (Kozlowski et al., 1999) and the input-mediator-output-input (IMOI) model of team effectiveness (Ilgen et al., 2005; Rosero et al., 2021), this study places emphasis on one period, namely input-mediator-output (IMO). *Mediator* expands on the number of variances by replacing *process* (Ilgen et al., 2005). The theoretical framework contains three parts and is shown in Fig. 1, accompanying the main hypotheses of this study.

Among the individual characteristics included in *input* are gender, education degree, field of education, family entrepreneurial history, and learner’s prior entrepreneurial experience. ICT, virtual teamwork, and taskwork are mediators. The output or performance section discusses the effectiveness of virtual team learning for EC.

### Hypotheses

The improvement of cognitive competence requires interaction between teachers and learners (Agudo-Peregrina et al., 2014). Entrepreneurial educators focus on constructivist learning theory and “learning by doing” by providing EE programs or projects (Bell & Bell, 2020; Hytti et al., 2010; Taylor & Thorpe, 2004). Little, however, is known about the attitude toward the effectiveness of online learning from students’ sides (McConnell, 2018), especially in EE. Both teamwork and taskwork have distinct functions in EC, and their correlation needs to be compared. Additionally, the effectiveness of ICT applied in a virtual team is discussed below.

Problem-based learning (Igwe et al., 2021; Santateresa, 2016), project-based learning (Arias et al., 2018), and program-based learning (Duval-Couetil & Shartrand, 2016), all of which require team-based learning and teaching activities, encourage collaboration and cooperation. In other words, the methods mentioned are different organizational forms of team tasks. Taskwork is one critical element of team learning, providing diverse activities and tasks to help acquire entrepreneurial skills and mindsets. Two types of virtual tasks are common, the first, in the primary phase, of placing tasks into virtual environments. Learners might submit an authentic business plan or present the result through digital ways after completing face-to-face tasks of teams. A further type is totally virtual. An example of this is the completed virtual collaborative writing task that is processed in an online environment (Mayordomo & Onrubia, 2015). The difference between virtual entrepreneurial tasks and face-to-face tasks lies in whether participants adopt technical devices or introduce a virtual environment. At present, virtual tasks are combined with artificial intelligence and other cutting-edge technologies (van Ginkel et al., 2019; Wang et al., 2020). For example, gamers discover venture opportunities with other avatars and earn virtual currency in Second Life serious games. The effect of virtual tasks is complicated. Previous research showed online learning activities can facilitate learners’ knowledge and skills (Hart et al., 2019; Pei & Wu, 2019). Thus, the feedback and formative evaluation of virtual team activities are positive (Clark & Gibb, 2006). Similarly, virtual tasks probably affect the performance of virtual team learning for EC. Hence, we assumed this hypothesis:

#### Hypothesis 1

Virtual taskwork has a positive effect on the effectiveness of virtual team learning for entrepreneurship competence.

Entrepreneurial teammates, often with diverse backgrounds, who are both learning partners and co-founders, often develop teamwork, relationship, and social network skills, through their shared learning goals and work in a democratic, trusting, and safe environment (Harms, 2015). Teamwork is crucial for starting a business (Warhuus et al., 2017). Both taskwork and teamwork have been clearly researched in a face-to-face environment (Lepine et al., 2000). Task- and teamwork are discussed much more offline, than in virtual teams. Successful teamwork or team relationships enables teammates to improve their potential performance and satisfaction, as well as preventing conflicts and freeriding (De Dreu & Weingart, 2003; Scott et al., 2019). Therefore, team relationships affect team learning as well as teaching. Although the discussion of virtual teamwork is still ongoing, scholars argue that virtual team relationships are as good as in the face-to-face environment (Rogers & Lea, 2005), because they enable course attendees’ to overcome their feelings of disconnectedness and separation (Parrish et al., 2021). Hence, virtual team relationships might affect the effectiveness of virtual team learning in EE. The correlation between virtual team relationships and virtual team learning is discussed below.

#### Hypothesis 2

Virtual teamwork has a positive effect on the effectiveness of virtual team learning for entrepreneurship competence.

In addition, ICT impacts virtual team learning (Bell & Kozlowski, 2002; Carlson et al., 2013). In an EE setting, the utilization of this technology is a moderator between entrepreneurial intention and risk (Bandera et al., 2018). Web 2.0 technology can increase absorptive capacities, which have a positive effect on the social entrepreneurship behavior (García-Morales et al., 2020). At present, educational technology has a wide range of functions, from is sharing resources (the version of the text, audio, and video), and enabling assignments to be posted, to allowing exchanging ideas on forums or in social media groups. Learners and instructors are in the same social software networking groups, enabling them to inspire each other through discussion and communication. Meanwhile, every team has its own social media collaborative group to help them accomplish tasks. Teammates focus on the given tasks and share information in their social media group. Social media provide further opportunities for remote participants to connect and learn how to collaborate with others. In addition, ICT as a part of the virtual team learning method might have affected EC. Accordingly, *it is expected that virtual team technologies have a positive influence on EC* (Hypothesis 3).

#### Hypothesis 3

Information and communication technology has a positive effect on the effectiveness of virtual team learning for entrepreneurship competence.

Finally, this study takes into consideration individual characteristics or learners’ demographic backgrounds that affect team process and influence team performance (Entin & Serfaty, 1999), without considering the characteristics of groups. Based on previous academic studies and the practical experience of teachers, gender, education degree (Paray & Kumar, 2020), education field (Pittaway & Edwards, 2012), family entrepreneurial history (Nowiński et al., 2019; Wadhwa & Aggarwal, 2009), and prior entrepreneurial experience (Mathews & Moser, 1995; Ngoc Khuong & Huu An, 2016) influence entrepreneurial intention and learning. Hence, this study assumed that individual characteristics including gender, individual education degree, education field, family entrepreneurial history, and prior entrepreneurial experience affect virtual taskwork, virtual teamwork, and ICT separately.

#### Hypothesis 4a

Individual characteristics (gender, education degree, education field, family entrepreneurial history, and prior entrepreneurial experience) affect virtual taskwork.

#### Hypothesis 4b

Individual characteristics (gender, education degree, education field, family entrepreneurial history, and prior entrepreneurial experience) affect virtual teamwork.

#### Hypothesis 4c

Individual characteristics (gender, education degree, education field, family entrepreneurial history, and prior entrepreneurial experience) affect information and communication technology.

## Methodology

Given that self-perception has a strong bearing on actual competence (Mitchelmore & Rowley, 2010), the perception of participants was the main data source of the present study.

### Participants

A convenience sampling method was adopted. The four responding teachers applied virtual teams to their EE courses from three different level HEIs (a top university, a common university, and two vocational and technology HEIs), all of which are located in the Yangtze River Delta region, China. They distributed the online survey to the currently enrolled students. In the introduction letter of the survey, the authors emphasized that someone who has experience in social media groups and other kinds of virtual teams for entrepreneurial learning and teaching, is suitable to participate in the study. All responses were from HEIs. Initially, 707 respondents from HEIs completed the online survey and 682 valid responses were collected. Excluding two outliers (two and 100 years old) and seven missing responses or filled names, Min = 16, Max = 44, *M* = 19.68 years old, SD = 1.717. Education degree: Senior school or under (0.6%), three years college or vocational and technical education (38.3%), bachelor (60.3%), and master or over (0.9%). Science field contains social science (19.1%), nature science (17.7%), applied science (37.0%), formal science (13.2%), and humanities (13.0%). 27.7% of respondents had a family history of entrepreneurs and 62.3% did not. 9.2% of respondents had entrepreneurial experience and 91.8% did not.

### Design and instrument

The survey was designed and displayed using Microsoft Form by forwarding the link with a specific explanation in WeChat groups. This Chinese social media software is mainly for connecting with entrepreneurial teachers and learners, which is the most convenient and popular communication tool for collaboration with Chinese scholars. As a result of the number of active monthly accounts, responses reached 1.20 billion in the first quarter of 2020 (CAICT, 2020). The survey was designed, taking into account demography, 11 entrepreneurial competencies, teamwork, taskwork, and ICT. Beside of age, demographic questions took into account gender, educational field, education degree, history of family entrepreneurs, and prior entrepreneurial experience. Further questions included 11 related to competencies, seven related to virtual team learning, and one alternative question were included to test the hypotheses mentioned above. Researchers designed seven items related to the entrepreneurial *position*, “I can discover possible entrepreneurial opportunities.” and four related to *personality traits*, e.g., “I believe I can successfully start a valuable business.” Two items of taskwork (TA_EASE and TA_STRATEGE), two items of teamwork (TE_TRUST and TE_DURATION), and three of ICT (ICT_VARIOUS, ICT_FREQUENCY, and ICT_PROFICIENCY) tested taskwork, teamwork/ team relationship, and ICT (three main factors of the virtual team) separately, e.g., “I like tasks with moderate difficulty”, “After completing the group task, I still contact with the group members”, and “When I attend EE courses, I use ICT to communicate and discuss with teammates every time”. A seven-point Likert scale ranging from 1 = totally disagree to 7 = totally agree was applied to ensure structured answers. Adoption of this scale avoids Confucianism that emphasizes the *Golden mean*, not too much and too little (Niemiec, 2019), which might lead Chinese respondents to choose the middle answer. In terms of further discussion or collaboration, the alternative question was open and optional, such as *If you want to take part in further the research, please leave your e-mail address*.

### Procedure

When the first version of the survey was finished, four education technology experts and two teachers from entrepreneurship education provided feedback on the validity of content. The authors modified the survey and started a pilot survey among 72 participants who have experience with EE and were members of the same EE WeChat groups. The survey was then administered on a larger scale from 28 April to 30 June 2021. The two educators distributed the online survey to their students across their universities (one is a top-ranked HEI and one is a normal college). Additionally, students from three higher vocational education colleges answered the questions. The research team cleaned the data. Data analysis and discussion were conducted as shown below.

### Data analysis

The data analysis adopted IBM SPSS 28 software, which is a statistics analysis appropriate for social science. H1–H3 were tested using linear regression, with three elements of the virtual team used as the independent variables and the performance of virtual team learning for EC as a dependent variable. Analysis of variance (ANOVA) was adopted to test H4a, H4b, and H4c.

## Result

An alpha level of 0.05 was used for statistical tests. Except for demographic items, the research adopted the rest to factor analysis. Kaiser–Meyer–Olkin measure of sampling adequacy value is 0.959 > 0.9, Bartlett’s test of sphericity significance is 0.000 < 0.05. These items are quite suitable for exploratory factor analysis. Analysis used correlation matrix; the extraction method is principal component analysis; the rotation method is varimax with Kaiser normalization. Therefore, three factors with 11 competencies/items remained: Factor 1 *personality traits* including four items, Cronbach’s alpha = 0.879; Factor 2 *position* includes seven items, Cronbach’s alpha = 0.931; Factor 3 virtual team contains seven items, Cronbach’s alpha = 0.933; the alpha value of the overall formal items (except demographic questions) was 0.969, which proved all had an adequate level of inter-item reliability. The cumulative contribution rate of the interpretable variance of the survey sample is 76.022%. The descriptive statistics of 11 items of entrepreneurship competence is shown in Table 2.

**Table 2:** Descriptive statistics of 11 items of entrepreneurship competence

Section	Item	Mean	Std. deviation
Position	PO_FINANCE	5.30	1.145
	PO_MANAGE	5.48	1.012
	PO_RESORCE	5.33	1.134
	PO_OPPORTUNITY	5.17	1.297
	PO_ACTION	5.35	1.113
	PO_EXPERIENCE	5.56	0.980
	PO_NETWORK	5.57	1.090
Personality traits	TR_SELF-EFFICACY	5.37	1.088
	TR_PERSEVERENCE	5.49	1.084
	TR_RISK	5.65	1.007
	TR_CREATIVITY	5.40	1.128

### Hypothesis 1–3

The results of Hypothesis 1–3 are shown in Table 3. In general, the perception of *personality* is higher than the *position* in hypothesis 1–3 from virtual taskwork, teamwork, and ICT aspects.

**Table 3:** The results of Hypothesis 1–3

Hypothesis	Item	Mean	SD	Sig	Adjusted R^2^	B	Beta
Hypothesis 1 (Virtual taskwork)	Position	5.516	.904	< .001	.620	.710	.788
	Traits	5.504	.917	< .001	.654	.740	.809
Hypothesis 2 (Virtual teamwork)	Position	5.516	.904	< .001	.666	.753	.816
	Traits	5.504	.917	< .001	.711	.789	.843
Hypothesis 2 (ICT)	Position	5.516	.904	< .001	.691	.721	.767
	Traits	5.504	.917	< .001	.588	.792	.831

Descriptive statistics have been calculated for the ease of task (TA_EASE, *M* = 5.39, SD = 1.089), for strategy of completing task (TA_STRATEGE, *M* = 5.43, SD = 1.019), and for taskwork (*M* = 5.408, SD = 1.003). To investigate whether virtual taskwork has a positive on the effectiveness of EE (the *position* and *personality traits*), linear regression was applied, yielding results of *position*, *F*(1, 680) = 1114.107, *B* = 0.710, adjusted *R*^2^ = 0.620, *p* < 0.001, and *personality traits*, *F*(1, 680) = 1287.566, *B* = 0.740, adjusted *R*^2^ = 0.654, *p* < 0.001. Virtual taskwork positively affects both the *position* and *personality traits*. Therefore, hypothesis 1 is accepted.

Descriptive statistics have been calculated for trust each other (TE_TRUST, *M* = 5.63, SD = 1.028), the duration of the team relationship (TE_DURATION, *M* = 5.53, SD = 1.069), and teamwork (*M* = 5.577, SD = 0.981). To reveal the relationship between virtual teamwork and the effectiveness of EE, linear regression was used. The result showed virtual teamwork positively affects the *position*, *F*(1, 680) = 1357.979, *B* = 0.753, adjusted *R*^2^ = 0.666, *p* < 0.001, and *personality traits*, *F*(1, 680) = 1674.032, *B* = 0.789, adjusted *R*^2^ = 0.711, *p* < 0.001. Virtual teamwork or team relationship significantly affects EE in terms of the position and personality traits. Therefore, hypothesis 2 is accepted.

Various technologies (ICT_VARIOUS, *M* = 5.55, SD** = **1.015), the frequency of usage of ICT (ICT_FREQUENCY, *M* = 5.42, SD = 1.095), the proficiency of usage of ICT (ICT_PROFICIENCY, *M* = 5.46, SD = 1.091), and ICT (*M* = 5.475, SD = 0.963) have been calculated. Linear regression revealed that ICT is positive on the *position F*(1, 680) = 973.811, *B* = 0.721, adjusted *R*^2^ = 0.691, *p* < 0.001 and *personality traits F*(1, 680) = 1520.808, *B* = 0.792, adjusted *R*^2^ = 0.588, *p* < 0.001. ICT positively affects sub-EC: the *position* and *personality traits*. Therefore, hypothesis 3 is accepted.

Through multiple linear regression analysis with virtual teamwork, taskwork, and ICT as independent variables and *position* and *personal traits* as the dependent variable, in Eq. (1), *p* = 0.000 < 0.05, adjusted *R*^2^ = 0.760 and in Eq. (2), *p* = 0.000 < 0.05, adjusted *R*^2^ = 0.828 without collinearity in both equations.

1 $$Y_{{{\text{position}}}} = 0.398x_{{{\text{te}}}} + 0.301x_{{{\text{ta}}}} + 0.177x_{{{\text{ict}}}}$$

2 $$Y_{{{\text{traits}}}} = 0.374x_{{{\text{te}}}} + 0.258x_{{{\text{ta}}}} + 0.300x_{{{\text{ict}}}}$$

### Hypothesis 4a-c

The results of hypothesis 4a-c employing one-way ANOVA are shown in Table 4.

**Table 4:** The results of Hypothesis 4a-4c using ANOVA

Hypothesis	Characteristic	*F*	Sig
Hypothesis 4a	Gender	1.435	.231
	Education degree	2.807	.039
	Education field	.393	.814
	Entrepreneurial family history	6.807	.009
	Entrepreneurial experience	2.864	.091
Hypothesis 4b	Gender	4.174	.041
	Education degree	1.844	.138
	Education field	.335	.854
	Entrepreneurial family history	6.432	.011
	Entrepreneurial experience	3.401	.066
Hypothesis 4c	Gender	5.437	.020
	Education degree	4.246	.006
	Education field	.678	.608
	Entrepreneurial family history	6.077	.014
	Entrepreneurial experience	2.770	.096

ANOVA was used to test for the differences of demographics (gender, education field, entrepreneurial family background, education degree, and entrepreneurial experience) on the perception of virtual taskwork. Female and male students do not differ in terms of their perception of virtual taskwork for EE, *F*(1, 680) = 1.435*, p* = 0.231. Different education fields did not affect participants’ opinion, *F*(5, 677) = 0.393, *p* = 0.814. Moreover, whether learners’ family has their own business or not, this factor would not influence their opinion on virtual taskwork, *F*(1, 680) = 0.818, *p* = 0.366. However, a participant’s own entrepreneurial experience affected their attitudes toward virtual taskwork, *F*(1,680) = 6.807, *p* = 0.009. Further, the higher the education degree of learners, the higher score on the effectiveness of virtual taskwork, *F*(4, 678) = 2.807*, p* = 0.039. Therefore, hypothesis 4a is accepted for the education degree and prior entrepreneurial experience and rejected for gender, education field, entrepreneurial family background.

Additionally, ANOVA was used to test for differences in demographics (gender, education field, entrepreneurial family background, education degree, and entrepreneurial experience) on the perception of virtual teamwork. Higher or lower education degrees did not influence the perception of virtual teamwork, *F*(4,678) = 1.844*, p* = 0.138. Additionally, participants from social science, natural science, and the other three fields had similar attitudes toward virtual teamwork in this survey, *F*(5,677) = 0.335*, p* = 0.854. And there was no difference between learners with and without entrepreneurial experience when it came to virtual teamwork, *F*(1,680) = 3.401*, p* = 0.066. However, female participants rated higher than those of males, *F*(1,680) = 4.174*, p* = 0.041. Further, learners without entrepreneurial family backgrounds rated virtual teamwork higher than others, *F*(1,680) = 6.432*, p* = 0.011. Therefore, in Hypothesis 4b, gender and entrepreneurial family background are accepted, and education field, education degree, and entrepreneurial experience are rejected.

Further, ANOVA was used to test for the differences of demographics (gender, education field, entrepreneurial family background, education degree, and entrepreneurial experience) on the perception of ICT. There was no significant difference between education fields and the perception of ICT, *F*(5,677) = 0.678*, p* = 0.608. In addition, entrepreneurial experience was not an impact variable at this point, *F*(1,680) = 2.770*, p* = 0.096. However, gender did impact the attitudes of participants, *F*(1,680) = 5.437*, p* = 0.020. Moreover, their entrepreneurial family background affected the perception, *F*(1,680) = 6.077*, p* = 0.014, and the higher the education degree, the higher score on the effectiveness of ICT, *F*(1,680) = 4.246*, p* = 0.006. Therefore, in hypothesis 4c, gender, entrepreneurial family background, and education degree affect ICT. Education field and prior entrepreneurial experience are rejected.

## Discussion

Face-to-face team learning is still mainstream in Chinese HEIs, although leading Chinese universities create MOOCs (Massive Open Online Courses) on iCourse and XuetangX. Therefore, common pedagogical practice for virtual team learning is combined with a face-to-face environment to ensure learners’ learning success. This study assumed that the performance of virtual team learning was affected by virtual teamwork, taskwork, and ICT separately, as well as their interactional impacts. Additionally, the probable influence of five demographic factors on virtual teamwork, taskwork, and ICT was assessed.

### Findings of virtual taskwork and the impact of demography

There is a lack of guidance from instructors and a lack of practical learning experience, when it comes to the completion of virtual tasks. At the same time, entrepreneurial tasks involve aspects of business administration, finance, law, and other related knowledge and skills, requiring participants professional in both their specific areas (depth) and other disciplines (width), namely T-shaped talents or enterprisers (Demirkan & Spohrer, 2015; Chan et al., 2020). Therefore, educators provide support for potential entrepreneurs in order that they do not have to complete tasks alone, when they lack experience in a virtual learning environment. Findings indicate that virtual taskwork impacts the effectiveness of EE in terms of entrepreneurial *position* and *personality traits*, in particular, during the completion of virtual tasks,

In terms of *position*, learners might acquire competencies in finance, management, learning from experience, social networking, identifying opportunities, and taking action. Recognition of entrepreneurial opportunities is a critical and complicated skill for future entrepreneurs. The ability to identify entrepreneurial opportunities requires three antecedents: schematic (or mental frameworks) richness, schematic association, and schematic priming, coming from entrepreneurial expertise, practice, and intention (Valliere, 2013). Furthermore, awareness of opportunities, based on prior knowledge or information related to a specific industry or target customers (Baron, 2006). Moreover, spotting an opportunity requires innovation or creativity skills, facilitated through orchestrating resources (Andersén & Ljungkvist, 2021). Although learners and instructors expend much effort and attention on identifying opportunities, the learning effect is still lower than expected when it comes to awareness of opportunities in starting a business. Only a minority understands the complexity of both mastering industry trends and being aware of customers’ real needs. Mobilization of human, capital, or information resources pushes boundaries when completing tasks in a virtual team, along with the benefits of fewer costs and time than the face-to-face team (Barnowska & Kozaryn, 2018). It has been proven that taking the entrepreneurial initiative to integrate resources is complicated. For one thing, discovering the clients’ real needs should provide insight into the internal and external marketing environment, e.g., Porter's five forces model. At the same time, although learners may come up with an ideal entrepreneurial project, they need assistance in terms of capital, technology, or human resources, predicting the gestation activities of firms (Davidsson & Honig, 2003). Nascent entrepreneurs lack social and capital resources, compared with the experienced. Teammates apply knowledge of management and finance to collective tasks, the application being better understanding and remembering knowledge basis on Bloom’s Taxonomy. And problem-solving inspires new ideas, especially when instructors encourage learners to apply new methods/tools, and another perspective (Guest & King, 2004). Therefore, virtual taskwork can facilitate the entrepreneurial competence of *position*, the sub-entrepreneurial competency.

Although *personality traits* related to entrepreneurs are hard to acquire in a short time, virtual taskwork affects these traits, namely perseverance, self-efficacy, coping with ambiguity and risk, and creativity. For example, completion of intentional assignments develops self-confidence and tenacity, leading to self-efficacy and perseverance (Olson, 2017). Hence, educators assign tasks for learners, considering learners’ motivation and initiation. Through completing virtual taskwork together, teammates get to know and support each other.

The other findings are the effectiveness of the demographic items on the perception of virtual taskwork. Different education degrees and the presence/lack of entrepreneurial experience influenced participants’ virtual teamwork. In order to complete entrepreneurial tasks, teammates need to master their knowledge and competence in specific disciplines, where learners with a higher educational background perform better than the less educated. Students with a family business background learn from observation and are directly or indirectly influenced by family experience when they adopt business strategies.

### Findings of virtual teamwork and the impact of demography

Teamwork or team relationships in a virtual team environment come from various team activities organized by course designers. These activities facilitate teammates’ sharing cognition or knowledge, valuable for team performance or effectiveness, explained by shared mental models theory (Cannon-Bowers & Salas, 2001) according to which a cohesive team is formed through trust and a friendly team relationship (Salas et al., 2015). Team cohesion and team openness is positively related to team performance, moderated by the experience of communication media in a virtual team (Carlson et al., 2013). In this survey, virtual teamwork impacts the effectiveness of EE in terms of entrepreneurial *position* and *personality traits*. Hence, teamwork or team relationship has a causal association with the effectiveness of team learning.

When teammates work close together, they are more likely to be able to identify opportunities, learn from others’ experience, and acquire financial, management, and social networking skills. It might be that team cohesion and team personality impact directly on teammates’ *personality traits*, indirectly affecting entrepreneurial positional competencies through their emotion and motivation (Molleman, 2005). In the *position* section, both team cohesion and openness are vital for sharing information and exchanging ideas to identify entrepreneurial opportunities and mobilize resources in a virtual entrepreneurial team. When adopting a virtual team method, participants should pay attention to the relationship amongst teammates by increasing interest in learning content, solving conflicts, and improving trust, potentially extending their social networking across the world. The establishment of good team relationships will lead to teammates sharing more personal information and engaging in conversation. In a friendly environment, participants will subconsciously learn from peers, and their attitudes and behaviors influence the team atmosphere. Nevertheless, one feature of a friendly team atmosphere is mutual support—all for one and one for all (Baruch & Lin, 2012)—with teammates working together to solve problems. Furthermore, perseverance may also increase in a friendly and close environment. Where teammates are open, trust each other and work cohesively (Goldstein & Gafni, 2019), learning improves and the completion of collaborative tasks is facilitated (Xie et al., 2019). In the *traits* section, self-efficacy is a synonym of perceived control. Team cohesion positively affects perceived control in the online environment (Zhao et al., 2021). Thus, teamwork influences self-efficacy in a virtual team. Risk-taking or tolerance of ambiguity is the result of perceived control. *Creativity* comes with an environment where everyone shows their opinions freely and respects others’ ideas. Therefore, teamwork facilitates the *position* and *personality traits* of EC.

When it comes to demographic factors, various observations can be made. Around two-thirds (62.3%) of respondents have no entrepreneurial family background, meaning they have only theoretical or academic knowledge on entrepreneurial learning or teaching. The family background might be more beneficial for entrepreneurial practice in real marketing. In comparison, academic or school settings provide robust training when it comes to basic entrepreneurial knowledge and skills. Therefore, family education might affect entrepreneurial intention and practical competence, leading to a better team relationship. When female learners take on the role of manager, they facilitate collaboration in a team and the gender composition of online learning impacts the team performance (Beddoes & Panther, 2018; Song et al., 2015). Kariv et al. (2019) found that while experienced entrepreneurial learners prefer academic projects and nascent/wanna-preneurs might choose non-academic projects that focus on funding, marketing, etc., neither entrepreneurial experience nor educational level or field affected virtual teamwork/team relationships.

### Findings of ICT and the impact of demography

Remote teammates share opinions and experiences through threaded asynchronous discussion (Jeong & Hmelo-Silver, 2016; Warkentin & Beranek, 1999) and a synchronous ideas exchange, contributing to task completion and a close team relationship. There is a significant difference between ICT and the performance of virtual team learning in both *position* and *traits* section, being one critical aim of this survey.

In detail, the social software networking group provides asynchronous (text, audio, or recorded-video) or synchronous (real-time audio or video) methods to successfully discuss and exchange ideas for completing missions, probably extending participants’ social network. During the process, teammates’ or peers’ opinions probably inspire others (brain-storming), broadening their minds and presenting innovative views. Synchronous meetings help classify misunderstandings, assist struggling teammates, and reemphasize shared goals (Olson-Buchanan et al., 2020). Additionally, ICT devices enable permanent storage and access to all dialogues, learning materials, and other documents on every participant’s device, in compliance with data protection regulations. Learners and educators instantly review all recorded course-related information to increase the frequency and possibility of communication and learning success. On the one hand, social media software can merge with other software. For example, business canvas applications can plug in the learning groups. ICT provides more connective and collaborative possibilities because of the two features of usefulness and ease of use (Venkatesh & Davis, 2000). On the other hand, as teammates enjoy close collaboration and relationships, the learning atmosphere is more supportive and friendlier, facilitating intrinsic and extrinsic learning motivation as well as perseverance. Other *personality traits* related to EC also can be positively affected, with Web 2.0 and 3.0, for example, increasing *creativity*. The technology effortlessly combines with other cutting-edge technologies to enhance the quality of cooperation and even closer team relationships. Hence, ICT impacts the effectiveness of virtual team learning in EE.

Demographical findings showed that entrepreneurial family backgrounds, gender, and education degree impact the perception of ICT in EE. Participants with entrepreneurial family backgrounds commented less on the effectiveness of ICT. Luo et al. (2012) proved that business ties or Guanxi ties are more indispensable for organizations in Mainland China than for those overseas. In the Chinese business environment, business ties or Guanxi ties are based on fair exchanges and the principle of reciprocity. Enterprisers mainly cooperate with family members, good friends, and acquaintances on the periphery (Burt & Burzynska, 2017). In other words, their connections are often established through practical business or trade-based activities, which are not easy to acquire via virtual tools (Turnbull et al., 2021). Participants with entrepreneurial family backgrounds have more social ties, giving them access to resources and the latest industry information. It has been investigated whether ICT is beneficial for entrepreneurial activities. For example, ICT provides female entrepreneurs with connections to stakeholders in developing countries (Venkatesh et al., 2017). Participants whose family members own a business are better placed, in terms of methods and resources, to acquire EC than others. Female learners’ ICT literacy is slightly higher than that of males (Siddiq& Scherer, 2019), which could explain the reason for differing comments. Universities students scored higher than those from three-year college ones, namely education impacts the effectiveness of ICT (Paray & Kumar, 2020). In addition, the function of ICT is a communication tool that needs to be combined with other approaches. Hence, ICT reveals that educators can advance the acceptance of virtual team learning through attaching technologies in EE courses.

### Findings on the correlation of three factors

Multiple linear regression analysis explains how virtual taskwork, teamwork, and ICT might operate or affect the performance of virtual team learning in EE. This study attempts to explore the interrelationships between the three elements of virtual team learning. Based on the correlation coefficient, technology possessed a higher correlation with both teamwork (0.758) and taskwork (0.753) than the correlation between teamwork and taskwork (0.722) (see Eqs. 1, 2). Taskwork and teamwork are two aspects of team activities or behaviors in a face-to-face learning environment. The online or blended learning environment heavily depends on technical tools or devices for discussion and communication. ICT affects teamwork, e.g., when to use ICT and how to use it affect team relationships (Parrish et al., 2021). When learners are accustomed to and have sufficient skills in information technology, they can probably accept virtual team learning quickly, complete tasks successfully and efficiently (taskwork), leading to imitative team learning relationships with teammates (teamwork). Hence, the influence of technology needs to be considered when adopting virtual team learning.

In this study, virtual teamwork is the most important factor in equations of both *personality traits* and *position*. In other words, the performance of virtual team learning depends largely on the relationship among teammates and their ability to discuss and complete tasks. In addition, social media software is taken as a learning management system to distribute documents and announce related information in EE courses. This function of social media was adopted in particular by many Chinese educators who rarely, if ever, applied official learning management systems to their daily teaching, learning, and classroom management. One reason is that educators need to spend a lot of time on virtual groups, especially in large classes with over 40 students (McConnell, 2018). The student–teacher ratio for undergraduates in Chinese HEIs, on the other hand, was 17.4:1 in 2018 (MEPRC, 2019). ICT saves time for educators by organizing common questions and queries and improving learners’ well-being and academic performance (Samad et al., 2019).

## Conclusion and further research

Under the teacher-centered circumstances and lack of learners’ independence in the Chinese HEIs classroom (Yin et al., 2014), the approach to virtual team learning in campus-based universities is rarely adopted. Scholars, educators, policy-makers, and stakeholders need to get feedback and collect data from instructors and learners. The outcome of virtual team learning from entrepreneurial learners was collected and analyzed in this research.

The team learning method encourages learners to share information and resources (Gikas & Grant, 2013). Taskwork, teamwork, and ICT are three critical mediators of virtual team learning, affecting the performance of virtual team learning for EC in both *personality traits* and *position*. Compared with virtual taskwork and ICT, teamwork is the most critical factor in virtual team learning for EC. To improve the effectiveness of virtual team learning, scholars, educators, and policy-makers need to focus on these three factors, especially teamwork.

Meanwhile, academic researchers argue that learners’ backgrounds influence the performance of EE. This study statistically proved that both education degree and family entrepreneurial background do affect virtual teamwork and ICT. Additionally, females and males are different on ICT. Furthermore, gender and entrepreneurial family history affect taskwork in this study. Therefore, instructional designers should consider gender, education degree, and family entrepreneurial background when implementing an entrepreneurial course by the adoption of virtual team learning.

This current study shows several main limitations that subsequently need to be solved and discussed. The variances are discussed in general, including teamwork, taskwork, and ICT, as well as five demographic moderators. When collecting data, responses’ answers are extreme, namely all items are “totally agree” or “totally disagree”. One entrepreneurial teacher told researchers: “I totally agree on each item. I really want to use educational technology.” But others’ reasons cannot be collected by this questionnaire survey. The final question was aimed at acquiring an email address in order to further interview those interested. Chinese participants, however, prefer social media to email. Seventy-two responses filled in email addresses correctly and received emails from researchers, while only one response contacted the research team via WeChat. Hence, this study cannot gain further information related to structured answers. Subsequent research will avoid this question and may interview no less than 30 EE experts. In addition, all items are perceived from an individual aspect. Further research might analyze the perception from both individuals and teams (Zhao et al., 2021). Although this questionnaire survey method can collect data from a broad spectrum, it might disturb validity. To be more concise, however, the impact factors are complicated and many factors need to be controlled. An experimental setting might be adopted in further research to avoid internal and external validity threats. Since the responses from various HEIs located in the developed area (Yangtze River Delta region) of China, they do not represent the general situation in the whole of China. Future research might compare results with Germany and China, utilizing interviews or collecting data from other Chinese areas for further research.

## Appendix

Questionnaire of the perception of the effectiveness of virtual team learning for entrepreneurship competence.

**Table:**

Category	Items
Demography	Gender: Male/Female
	Age:
	Education: Senior school (secondary vocational school) or under, Vocational or three-year college, Bachelor, Over bachelor
	Education field: Social sciences (e.g., Economics, Law, Education), Natural sciences (e.g., Physics, Biology, Chemistry), Applied sciences (e.g., Civil Engineering, Applied Mathematics), Formal sciences (e.g., Mathematics, Statistics), Humanities sciences (e.g., Literature, Philosophy, History)
	There are self-employees in my family (parents and siblings): Yes/ No
	I have entrepreneurial experience or I am an entrepreneur: Yes/No
Entrepreneurship Competencies (Totally disagree-totally agree 7-point Likert scale)	I can discover possible entrepreneurial opportunities
	I am good at integrating and using the resources I need
	I can complete the task according to the plan
	I often have new or unique ideas
	I know how much money is needed to start a company
	I will make a plan to achieve a goal
	I believe I can successfully start a valuable business
	In working with others, I can establish good relationships with others
	When encountering problems, I can actively face and solve them
	I can learn from my prior experience
	When I meet problems, I don't give up easily
Virtual Team (Totally disagree-totally agree 7-point Likert scale)	I like tasks with moderate difficulty
	I can adopt appropriate strategies or methods to solve the problem
	Various ICT methods have different functions
	Online cooperation has helped me and my team members build a relationship of mutual trust and common goals
	After completing the group task, I still contact with the group members
	When I attend EE courses, I use ICT to communicate and discuss with teammates every time
	I can use a variety of ICT methods proficiently
